# Supplementary material for: Projected climate-driven changes in pollen emission season length and magnitude over the continental United States
Source: Nat Commun. 2022 Mar 15;13:1234. doi: 10.1038/s41467-022-28764-0 (PMC8924258; doi:10.1038/s41467-022-28764-0)
Supplement: Supplementary file 1 — Supplementary Information [file 41467_2022_28764_MOESM1_ESM.pdf]

Projected climate-driven changes in pollen emission season length and magnitude over the continental United States

Supplementary Information

Yingxiao Zhang[1] and Allison Steiner[1]

[1]Department of Climate and Space Sciences and Engineering, University of Michigan, Ann Arbor, United States

Supplementary Figures:

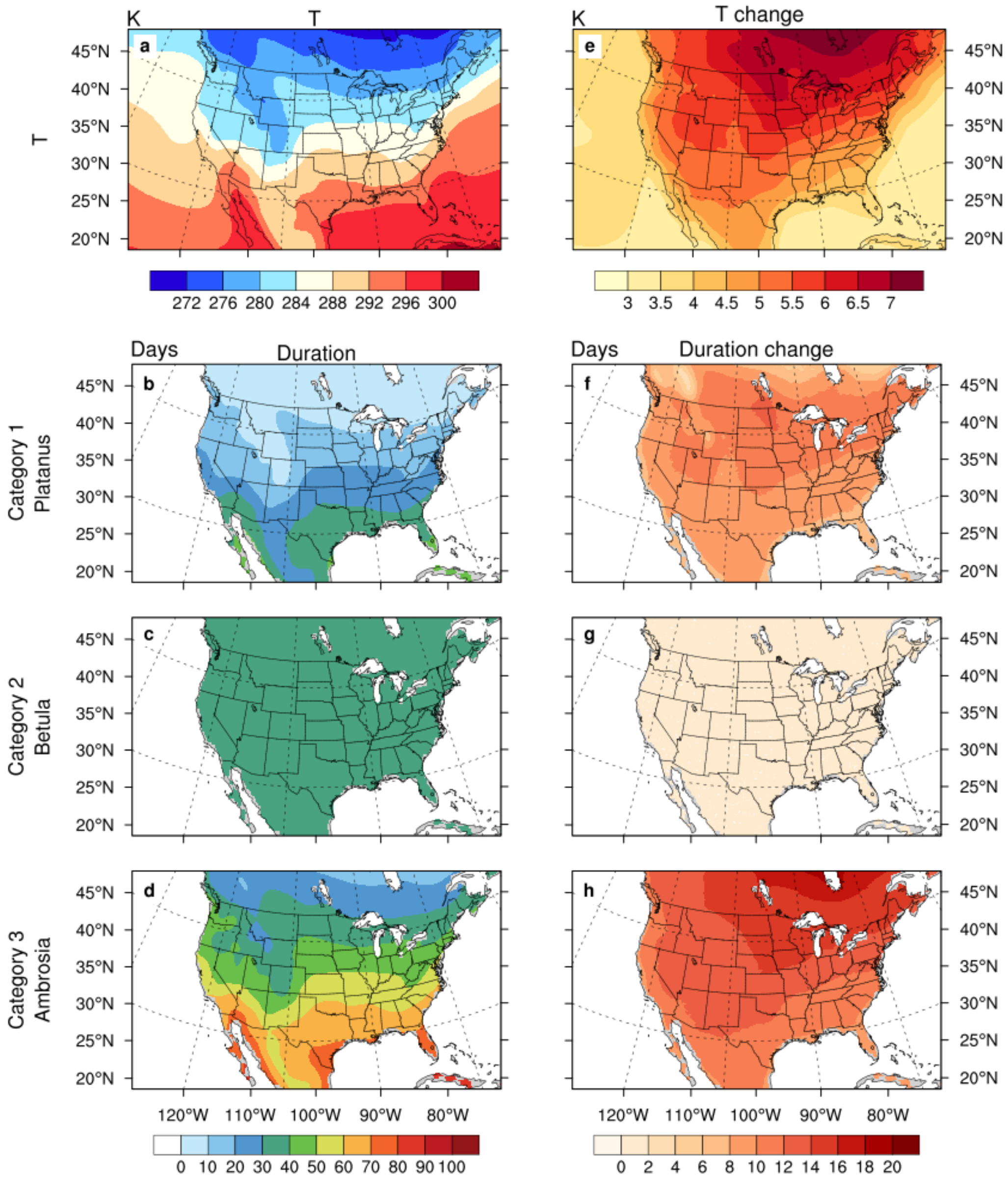

Figure S1 Pollen season duration and duration change for three categories
Multi-model averaged spatial distribution of historical (1995-2014) temperature (K) (**a**), historical pollen season durations (days) (**b-d**), and the end of the century (2081-2100) with scenario SSP585 temperature change (**e**), and pollen season duration (**f-h**)). One vegetation type is selected to represent each of the three categories of pollen season duration change (Figure 2): **b, f**, *Platanus* (Category 1); **c, g**, *Betula* (Category 2); **d, h**, *Ambrosia* (Category 3).

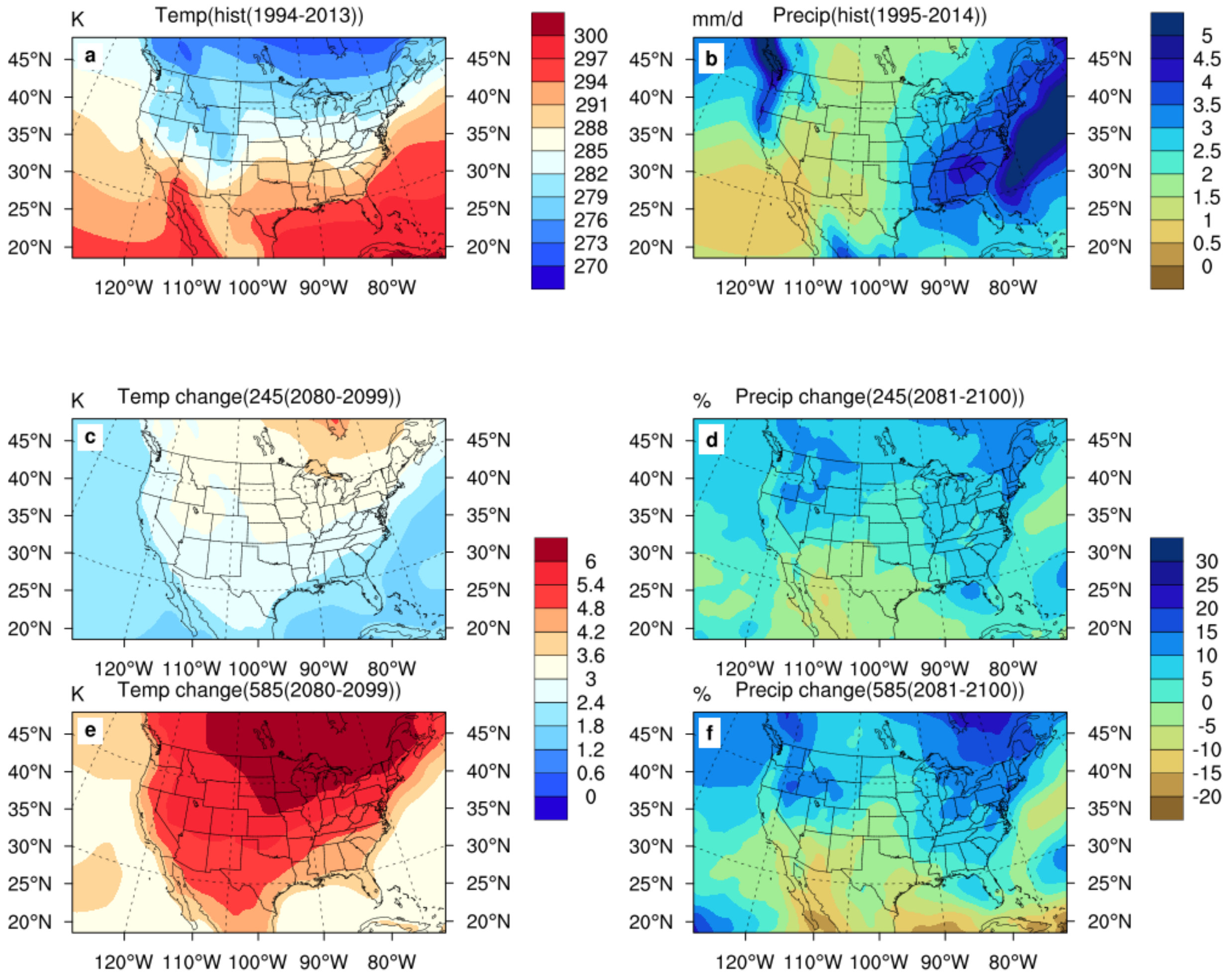

Figure S2 Future temperature and precipitation from CMIP6 models

Multi-model annual mean temperature and precipitation over the continental U.S. (20-year average). (**a, b)** historical (1995-2014) distributions for temperature (K) (**a**) and precipitation (mm/d) (**b**) and (**c-f),** future (2081-2100) changes for temperature (K) (**c, e**) and precipitation (%) (**d, f**) under scenario SSP 245 (**c, d**) and SSP 585 (**e, f**).

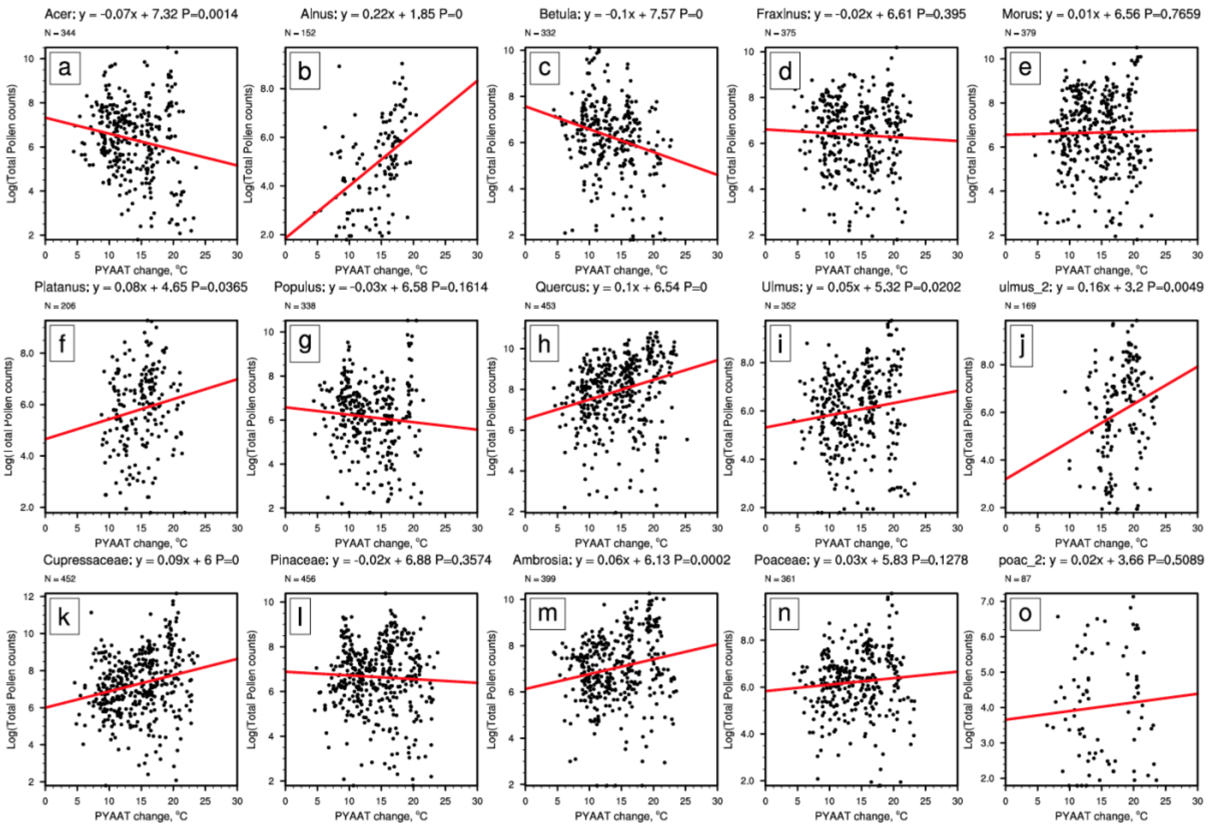

Figure S3 Relationships between annual total pollen counts and temperature.

Linear regressions for log-transformed accumulated annual total pollen counts versus previous-year annual average temperature (PYAAT, °C) for all taxa. Each point signifies one station per year from 2003 to 2010, with N as the total number of observations utilized in the regression. Regression statistics are summarized in Table S1.

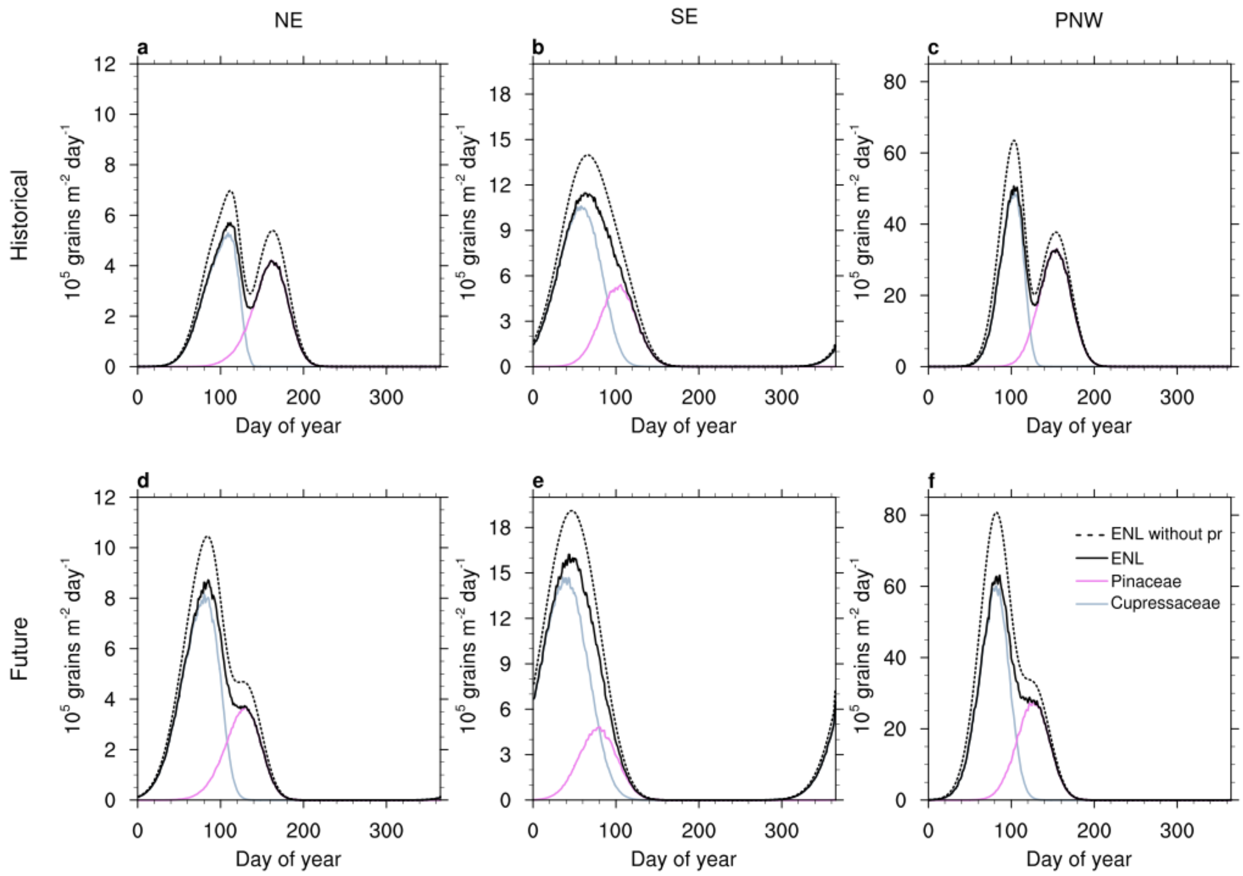

Figure S4 Evergreen Needleleaf (ENL) pollen season phenology

20-year multi-model average time series of daily pollen emission flux ($grains\ m^{-2}d^{-1}$) of 2 individual tree families that comprise ENL (Pinaceae and Cupressaceae) and the total ENL emission with and without precipitation. (**a-c**) historical (1995-2014) emissions and (**d-f**) emissions at the end of the century (2081-2100) for SSP 585. Columns from left to right: Northeast, NE (**a, d**); Southeast, SE (**b, e**); Pacific Northwest, PNW (**c, f**).

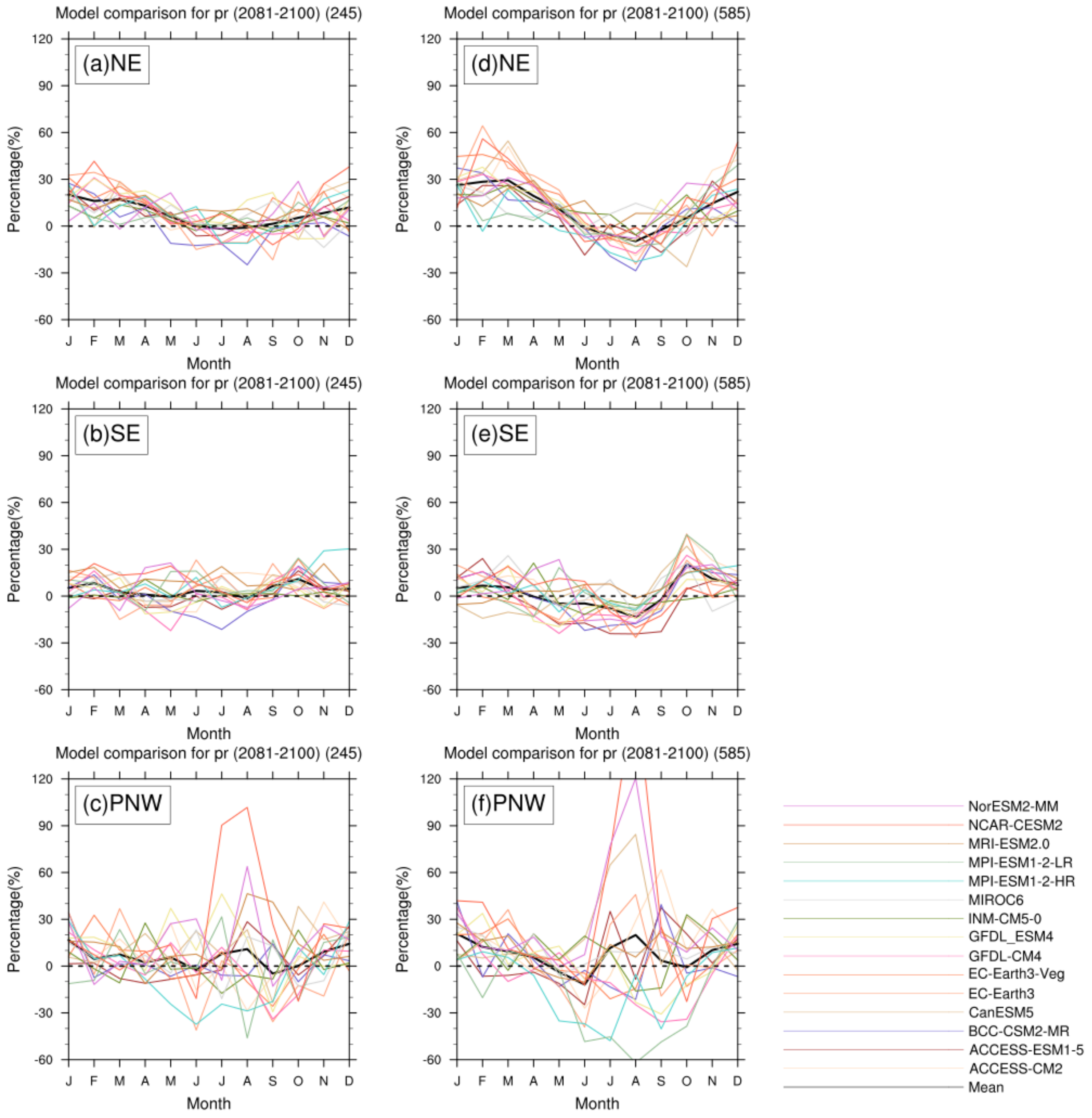

Figure S5 Seasonal precipitation changes in the future.

Spatially averaged change of 20-year monthly averaged daily precipitation for individual CMIP6 models and the multi-model mean in the Northeast region (**a, d)**, Southeast region (**b, e**), and Pacific Northeast region (**c, f**) at the end of century (2081-2100) under scenario SSP 245 (**a-c**) and SSP 585 (**b-f**) compared to historical period (1995-2014) (unit: percentage (%)). Black lines show the model mean. Region boundaries are shown in Fig. 2.

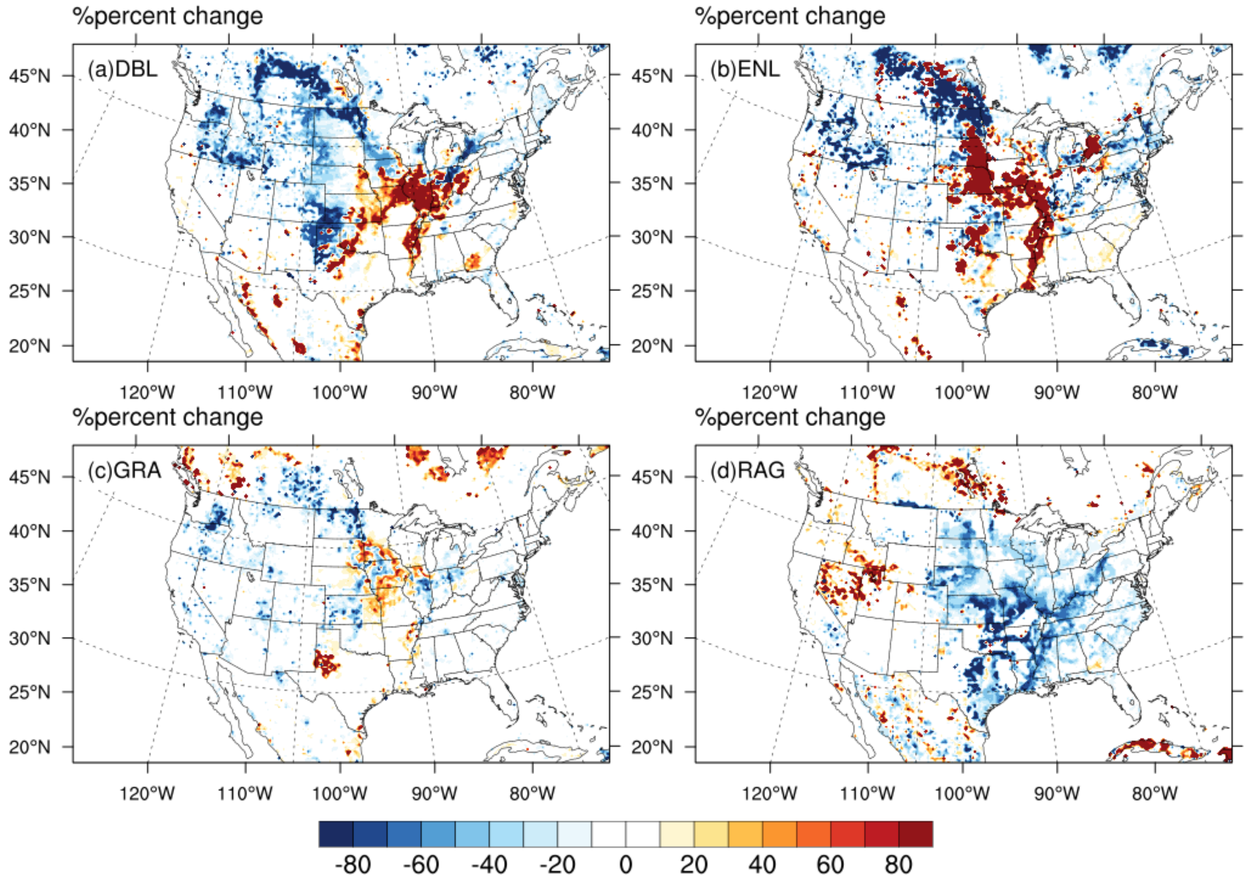

Figure S6 Future PFT Land cover change.

a-d, GCAM-Demeter (Chen et al.[1]) projected land cover change in the future (2100) compared to the historical (2015) for 4 PFTs: Deciduous Broadleaf (DBL) (**a**), Evergreen Needleleaf (ENL) (**b**), Grasses (GRA) (**c**) and Ragweed (RAG), which is calculated using the cropland and urban coverage (see Wozniak and Steiner[2]) (**d**) (unit: %).

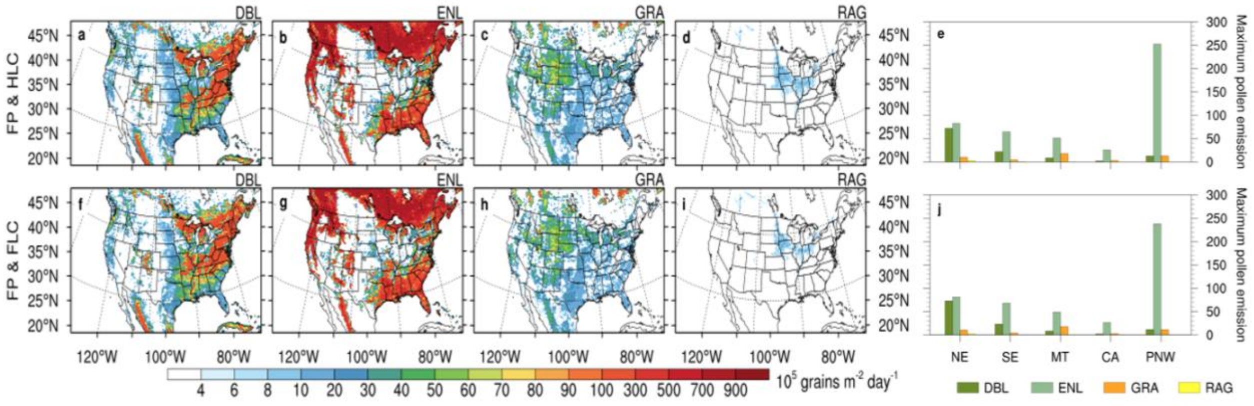

Figure S7 Simulated maximum daily pollen emission with historical and future land cover.

a-e, Future average (2081-2100) maximum daily pollen emission flux (FP) with historical (2015) land cover fraction (HLC) over the United States (unit: $grains\ m^{-2}d^{-1}$). **f-j**, same as **a-e** but using future (2100) land cover fraction (FLC). The simulation is conducted using PFT-based pollen emission model (PECM) and driving by multi-model average meteorology input data in at the end of the century (2081-2100). The percentage change of maximum daily pollen emission flux due to the future land cover change is shown in Fig.4 p-s in the main manuscript (change = FP & FLC – FP & HLC). Each column represents different PFTs, from left to right: DBL (**a, f**), ENL (**b, g**), GRA (**c, h**), RAG (**d, i**).

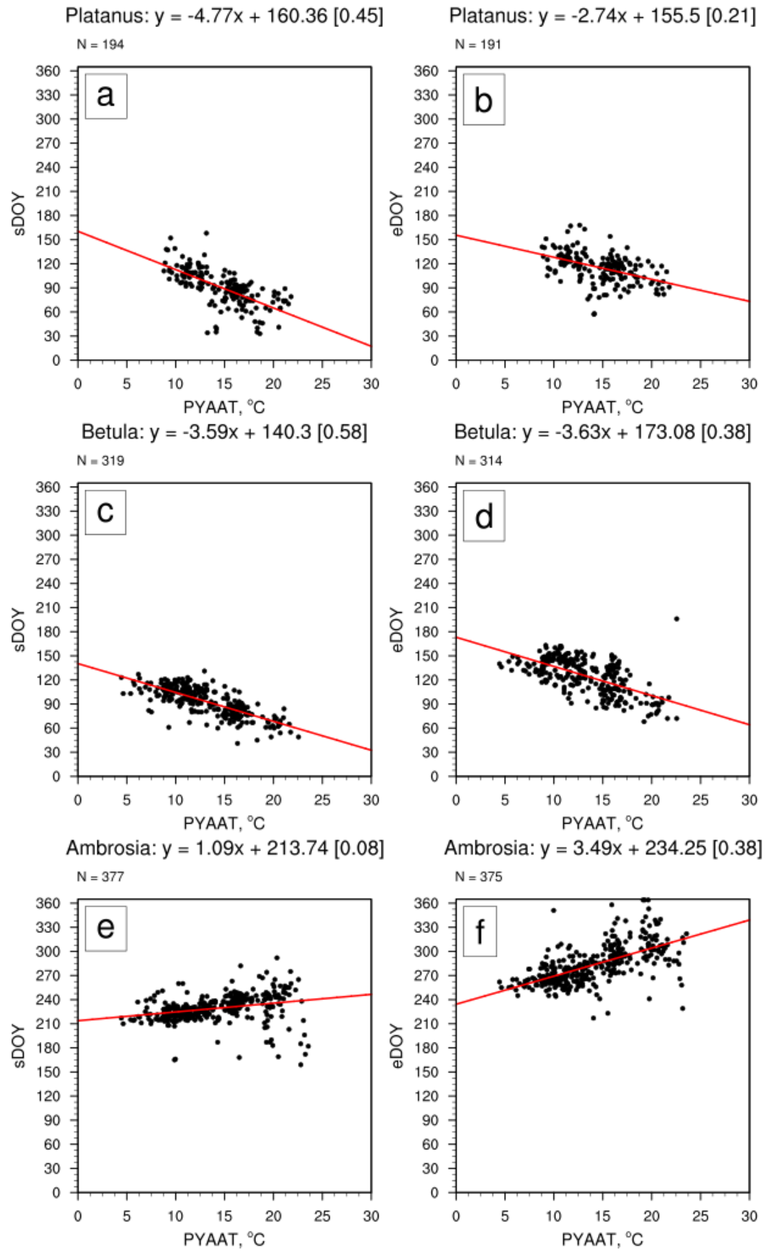

Figure S8 Linear relationship between observed pollen season timing and temperature.

a-f, Phenological regression for start day of year (sDOY; **a, c, e**) and end day of year (eDOY; **b, d, f**) versus previous-year annual average temperature (PYAAT, ℃). One vegetation type is selected to represent each of the three categories (shown in Fig. 1) of pollen season duration change: **a, b**, *Platanus* (Category 1); **c, d,** *Betula* (Category 2); **e, f,** *Ambrosia* (Category 3). Each point signifies one station per year from 2003 to 2010 with N as the total number of observations.

Supplementary Tables:

Taxon or PFT	P_{annual} 10^7 grains m^{-2} yr^{-1}	Reference for P_{annual}	m_{prod}	b_{prod}
Deciduous broadleaf forest (DBL)				
Acer	134	Katz et al. (2020), Tormo Molina et al. (1996)	-0.07	7.32
Alnus	210	Helbig et al. (2004)	0.22	1.85
Betula	115	Katz et al. (2020), Jato et al. (2007)	-0.1	7.57
Fraxinus	161	Tormo Molina et al. (1996)	-0.02	6.61
Morus	1260	Katz et al. (2020)	0.01	6.56
Platanus	392	Katz et al. (2020), Tormo Molina et al. (1996)	0.08	4.65
Populus	100	Katz et al. (2020), Tormo Molina et al. (1996)	-0.03	6.58
Quercus	143	Katz et al. (2020), Tormo Molina et al. (1996), Gmoez-casero et al. (2010)	0.1	6.54
Ulmus (late)	31.6	Katz et al. (2020),Tormo Molina et al. (1996)	0.05	5.32
Ulmus (late)	31.6	Katz et al. (2020),Tormo Molina et al. (1996)	0.16	3.2
DBL	283	This paper	N/A	N/A
Evergreen needleleaf forest (ENL)				
Cupressaceae	1050	Hidalgo et al. (1999), Damialis et al. (2011), Aboulaich et al. (2008)	0.09	6
Pinaceae	101	Tormo Molina et al. (1996)	-0.02	6.88
ENL	575.5	This paper	N/A	N/A
Grasses (GRA)				
Poaceae(C3)	46.2	Prieto-Baena et al. (2003)	0.03	5.83
Poaceae(C4)	4.62	Prieto-Baena et al. (2003)	0.02	3.66
Ragweed (RAG)				
Ambrosia	119	Fumanal et al. (2007)	0.06	6.13

Table S1 Production factor (P_{annual}) and production regression coefficients (m_{prod} and b_{prod}) for the 13 individual taxa and corresponding PFTs used in this study.

Production factors (P_{annual}) are based on literature value averages. Individual taxa and families are organized into four PFTs, with the two aggregated tree PFTs (DBL and ENL). Regression slope and intercept (m_{prod} and b_{prod}, respectively) used to derive the annual pollen production (pf_{annual}) as a function of temperature are provided. The taxa that have a negative correlation with temperature are colored with grey. Note the PFT-based model does not consider the change of pollen production with temperature and regression coefficients are not provided. The source data are provided in source data file.

Morris Index	parameters	Taxa														
		ACER	ALDR	BETU	CUPR	FRAX	MORU	PINU	PLAT	POPU	QUER	ULNU	ULN2	GRC3	GRC4	AMBR
μ*	a	9	9	9	9	9	9	9	9	9	9	9	9	9	9	9
	m_{sDOY}	8	3	8	7	7	8	8	8	7	7	8	2	8	3	8
	b_{sDOY}	7	8	7	4	4	7	6	7	5	8	7	6	6	8	6
	m_{eDOY}	6	1	6	8	8	6	7	4	4	6	6	4	7	6	7
	b_{eDOY}	5	7	4	6	6	5	5	2	2	5	5	5	5	7	5
	m_{prod}	4	5	3	5	5	3	4	5	8	4	4	3	4	1	4
	b_{prod}	3	2	2	3	3	4	2	6	3	3	3	1	3	2	3
	P_{norm}	2	4	1	2	1	1	1	1	1	2	1	7	2	4	1
	P_{annual}	1	6	5	1	2	2	3	3	6	1	2	8	1	5	2
σ	a	9	9	8	9	9	9	9	9	9	9	9	9	9	9	9
	m_{sDOY}	7	3	9	6	5	8	8	2	6	7	8	1	7	3	8
	b_{sDOY}	8	8	7	3	3	7	6	6	5	8	7	6	6	8	6
	m_{eDOY}	6	1	6	8	8	4	5	7	4	6	6	4	8	7	7
	b_{eDOY}	5	7	4	7	7	6	7	1	1	5	5	5	5	6	5
	m_{prod}	4	4	3	5	6	3	4	5	8	4	4	3	4	1	4
	b_{prod}	3	2	2	4	4	5	3	8	3	3	3	2	3	2	3
	P_{norm}	2	5	1	2	1	1	1	4	2	1	1	7	2	5	1
	P_{annual}	1	6	5	1	2	2	2	3	7	2	2	8	1	4	2

Table S2 Model sensitivity analysis parameter ranks from Morris indices.

The simulated Morris indices (mean μ^* and standard division σ) for the 9 input parameters for the 15 model taxa. The highest 4 ranking parameters are highlighted with warm colors, indicating a larger importance of the pollen emission simulation.

**Model**	**Atmospheric resolution (long x lat)**	**Modeling group**	**References**
**ACCESS-CM2**	1.875° × 1.25°, L85	CSIRO-ARCCSS	Dix et al. (2019)
**ACCESS-ESM1-5**	1.875° × 1.25°, L38	CSIRO	Ziehn et al. (2019)
**BCC-CSM2-MR**	1.125° × 1.125°, L46	BCC	Zhang et al. (2019)
**CanESM5**	2.81° × 2.81°, L49	CCCma	Swart et al. (2019)
**EC-Earth3**	0.7° × 0.7°, L91	EC-Earth-Consortium	EC-Earth Consortium (EC-Earth) (2019)
**EC-Earth3-Veg**	0.7° × 0.7°, L91	EC-Earth-Consortium	EC-Earth Consortium (EC-Earth) (2019)
**GFDL-CM4**	1.25° × 1°, L33	NOAA-GFDL	Silvers et al. (2018)
**GFDL-ESM4**	1.25° × 1°, L49	NOAA-GFDL	Horowitz et al. (2018)
**INM-CM5-0**	2° × 1.5°, L73	INM	Volodin et al. (2019)
**MIROC6**	1.4° × 1.4°, L81	MIROC	Takemura, Toshihiko (2019)
**MPI-ESM1-2-HR**	0.9375° × 0.9375°, L95	MPI-M DWD DKRZ	Jungclaus et al. (2019)
**MPI-ESM1-2-LR**	1.875° × 1.875°, L47	MPI-M AWI	Brovkin et al. (2019)
**MRI-ESM2.0**	1.125° × 1.125°, L80	MRI	Yukimoto et al. (2019)
**NCAR-CESM2**	1.25° × 0.9375°, L32	NCAR	Danabasoglu, Gokhan (2019)
**NorESM2-MM**	1.25° × 0.9375°, L32	NCC	Bentsen et al. (2019)

Table S3 CMIP6 models used in the study.

Modeling group information and spatial resolution are provided at https://wcrp-cmip.github.io/CMIP6_CVs/docs/CMIP6_source_id.html
with data citations in http://bit.ly/CMIP6_Citation_Search. CMIP6 data are regridded to a 25km Lambert Conformal Conic projection using the Earth System Modeling Framework (ESMF) higher-order patch regridding method over the United States to match the spatial resolution of PECM.

Name	Symbol	Units	Taxa	Lower limit	Upper limit
Gaussian width	a	N/A	All taxa	2.8	3.2
Slope of the regression between start date (sDOY) of pollen season and temperature	sDOY_m	Days $°C^{-1}$	ACER	-2.41	-1.47
			ALDR	-7.66	-4.44
			BETU	-3.96	-3.23
			CUPR	-6.45	-5.25
			FRAC	-5.15	-4.12
			MORU	-4.45	-3.67
			PINU	-6.46	-5.21
			PLAT	-5.56	-3.98
			POPU	-2.73	-1.78
			QUER	-4.53	-3.79
			ULNU	-5.12	-4.27
			ULN2	2.15	4.96
			GRC3	-5.38	-4.3
			GRC4	-1.18	1.12
			AMBR	0.7	1.48
Intercept of the regression between start date (sDOY) of pollen season and temperature	sDOY_b	Days	ACER	97.21	110.55
			ALDR	126.3	172.24
			BETU	135.3	145.3
			CUPR	129.13	146.66
			FRAC	140.8	155.93
			MORU	148.77	160.29
			PINU	168.26	186.82
			PLAT	148.41	172.31
			POPU	108.22	121.15
			QUER	142.84	153.95
			ULNU	124.97	137.27
			ULN2	153.39	204.46
			GRC3	177.38	193.27
			GRC4	228.17	264.53
			AMBR	208.05	219.43
Slope of the regression between end date (eDOY) of pollen season and temperature	eDOY_m	Days $°C^{-1}$	ACER	-2.28	-0.85
			ALDR	-5.58	-1.95
			BETU	-4.18	-3.08
			CUPR	-3.33	-2.1
			FRAC	-3.39	-2.44
			MORU	-3.53	-2.54
			PINU	-5.68	-4.47
			PLAT	-3.53	-1.95
			POPU	-0.91	0.38
			QUER	-2.48	-1.65
			ULNU	-3.08	-1.83
			ULN2	3.59	6.37
			GRC3	-1.85	-0.42
			GRC4	1.65	4.12
			AMBR	3.03	3.96
Intercept of the regression between end date (eDOY) of pollen season and temperature	eDOY_b	Days	ACER	126.68	146.4
			ALDR	109.36	161.39
			BETU	165.49	180.66
			CUPR	130.65	148.58
			FRAC	148.62	162.41
			MORU	167.15	181.77
			PINU	206.51	224.36
			PLAT	143.53	167.47
			POPU	110.19	127.6
			QUER	159	171.49
			ULNU	119.4	137.59
			ULN2	163.66	214.79
			GRC3	179.52	200.48
			GRC4	205.29	244.5

			AMBR	227.48	241.03
Slope of the regression between log-transformed annual total pollen counts and temperature	prod_m	Grains m⁻³ °C⁻¹	ACER	-0.12	-0.03
			ALDR	0.14	0.29
			BETU	-0.14	-0.05
			CUPR	0.05	0.13
			FRAC	-0.055	0.022
			MORU	-0.038	0.052
			PINU	-0.052	0.019
			PLAT	0.0049	0.15
			POPU	-0.081	0.014
			QUER	0.061	0.13
			ULNU	0.0079	0.093
			ULN2	0.048	0.27
			GRC3	-0.008	0.063
			GRC4	-0.049	0.098
			AMBR	0.031	0.098
Intercept of the regression between log-transformed annual total pollen counts and temperature	eDOY_b	$Grains\,m^{-3}$	ACER	6.7	7.95
			ALDR	0.71	2.99
			BETU	6.93	8.2
			CUPR	5.43	6.57
			FRAC	6.04	7.17
			MORU	5.89	7.22
			PINU	6.36	7.41
			PLAT	3.55	5.76
			POPU	5.93	7.23
			QUER	6	7.07
			ULNU	4.69	5.94
			ULN2	1.22	5.17
			GRC3	5.31	6.35
			GRC4	2.49	4.82
			AMBR	5.64	6.63
Parameter used to normalize the regression between log-transformed annual total pollen counts and temperature	Pnorm	$Grains\,m^{-3}$	ACER	142.12	1833.85
			ALDR	9.670855	502.5016
			BETU	133.77	1760.97
			CUPR	471.14	4700.54
			FRAC	188.85	1789.35
			MORU	208.0854	2908.632
			PINU	272.0069	2176.695
			PLAT	37.38186	2804.972
			POPU	115.9617	1691.528
			QUER	978.6627	7774.429
			ULNU	122.0912	1467.142
			ULN2	6.802754	8888.089
			GRC3	180.148	1429.732
			GRC4	5.918866	514.765
			AMBR	441.5792	3145.443
Pollen annual emission baseline	Pannual	$Grains\,m^{-2}$	ACER	4.92E+07	6.50E+09
			ALDR	6.86E+08	5.22E+09
			BETU	8.92E+08	1.40E+09
			CUPR	1.56E+09	4.35E+10
			FRAC	3.37E+08	3.20E+09
			MORU	4.12E+09	3.13E+10
			PINU	4.76E+08	1.32E+09
			PLAT	1.38E+09	5.72E+09
			POPU	6.82E+08	1.29E+09
			QUER	1.50E+08	6.10E+09
			ULNU	9.34E+07	7.86E+08
			ULN2	9.34E+07	7.86E+08
			GRC3	8.53E+07	8.39E+08
			GRC4	8.53E+06	8.39E+07
			AMBR	3.89E+08	2.96E+09

Table S4 Uncertainty ranges for input parameters for Morris sampling.

The uncertainty ranges of 9 input parameters for the 15 individual taxa. Using the Morris method, the input parameter sets for each model sensitivity run are sampled within the above ranges. The source data are provided in source data file.

Supplementary References:

1. Chen, M. *et al.* Global land use for 2015–2100 at 0.05° resolution under diverse socioeconomic and climate scenarios. *Sci. Data 2020 71* **7**, 1–11 (2020).
2. Wozniak, M. C. & Steiner, A. L. A prognostic pollen emissions model for climate models (PECM1.0). *Geosci. Model Dev* **10**, 4105–4127 (2017).

CMIP6 data citations:

Bentsen, Mats; Olivié, Dirk Jan Leo; Seland, Øyvind; Toniazzo, Thomas; Gjermundsen, Ada; Graff, Lise Seland; Debernard, Jens Boldingh; Gupta, Alok Kumar; He, Yanchun; Kirkevåg, Alf; Schwinger, Jörg; Tjiputra, Jerry; Aas, Kjetil Schanke; Bethke, Ingo; Fan, Yuanchao; Griesfeller, Jan; Grini, Alf; Guo, Chuncheng; Ilicak, Mehmet; Karset, Inger Helene Hafsahl; Landgren, Oskar Andreas; Liakka, Johan; Moseid, Kine Onsum; Nummelin, Aleksi; Spensberger, Clemens; Tang, Hui; Zhang, Zhongshi; Heinze, Christoph; Iversen, Trond; Schulz, Michael (2019). NCC NorESM2-MM model output prepared for CMIP6 CMIP. Earth System Grid Federation. doi:https://doi.org/10.22033/ESGF/CMIP6.506 .

Brovkin, Victor; Wieners, Karl-Hermann; Giorgetta, Marco; Jungclaus, Johann; Reick, Christian; Esch, Monika; Bittner, Matthias; Legutke, Stephanie; Schupfner, Martin; Wachsmann, Fabian; Gayler, Veronika; Haak, Helmuth; de Vrese, Philipp; Raddatz, Thomas; Mauritsen, Thorsten; von Storch, Jin-Song; Behrens, Jörg; Claussen, Martin; Crueger, Traute; Fast, Irina; Fiedler, Stephanie; Hagemann, Stefan; Hohenegger, Cathy; Jahns, Thomas; Kloster, Silvia; Kinne, Stefan; Lasslop, Gitta; Kornblueh, Luis; Marotzke, Jochem; Matei, Daniela; Meraner, Katharina; Mikolajewicz, Uwe; Modali, Kameswarrao; Müller, Wolfgang; Nabel, Julia; Notz, Dirk; Peters, Karsten; Pincus, Robert; Pohlmann, Holger; Pongratz, Julia; Rast, Sebastian; Schmidt, Hauke; Schnur, Reiner; Schulzweida, Uwe; Six, Katharina; Stevens, Bjorn; Voigt, Aiko; Roeckner, Erich (2019). MPI-M MPIESM1.2-LR model output prepared for CMIP6 C4MIP. Earth System Grid Federation. doi:https://doi.org/10.22033/ESGF/CMIP6.748 .

Danabasoglu, Gokhan (2019). NCAR CESM2 model output prepared for CMIP6 AerChemMIP. Earth System Grid Federation. doi:https://doi.org/10.22033/ESGF/CMIP6.2181 .

Dix, Martin; Bi, Doahua; Dobrohotoff, Peter; Fiedler, Russell; Harman, Ian; Law, Rachel; Mackallah, Chloe; Marsland, Simon; O'Farrell, Siobhan; Rashid, Harun; Srbinovsky, Jhan; Sullivan, Arnold; Trenham, Claire; Vohralik, Peter; Watterson, Ian; Williams, Gareth; Woodhouse, Matthew; Bodman, Roger; Dias, Fabio Boeira; Domingues, Catia; Hannah, Nicholas; Heerdegen, Aidan; Savita, Abhishek; Wales, Scott; Allen, Chris; Druken, Kelsey;

Evans, Ben; Richards, Clare; Ridzwan, Syazwan Mohamed; Roberts, Dale; Smillie, Jon; Snow, Kate; Ward, Marshall; Yang, Rui (2019). CSIRO-ARCCSS ACCESS-CM2 model output prepared for CMIP6 CMIP. Earth System Grid Federation. doi:https://doi.org/10.22033/ESGF/CMIP6.2281 .

EC-Earth Consortium (EC-Earth) (2019). EC-Earth-Consortium EC-Earth3 model output prepared for CMIP6 CMIP. Earth System Grid Federation. doi:https://doi.org/10.22033/ESGF/CMIP6.181 .

EC-Earth Consortium (EC-Earth) (2019). EC-Earth-Consortium EC-Earth3-Veg model output prepared for CMIP6 CMIP. Earth System Grid Federation. doi:https://doi.org/10.22033/ESGF/CMIP6.642 .

Horowitz, Larry W.; Naik, Vaishali; Sentman, Lori; Paulot, Fabien; Blanton, Chris; McHugh, Colleen; Radhakrishnan, Aparna; Rand, Kristopher; Vahlenkamp, Hans; Zadeh, Niki T.; Wilson, Chandin; Ginoux, Paul; He, Jian; John, Jasmin G.; Lin, Meiyun; Paynter, David J; Ploshay, Jeffrey; Zhang, Alex; Zeng, Yujin (2018). NOAA-GFDL GFDL-ESM4 model output prepared for CMIP6 AerChemMIP. Earth System Grid Federation. doi:https://doi.org/10.22033/ESGF/CMIP6.1404 .

Jungclaus, Johann; Bittner, Matthias; Wieners, Karl-Hermann; Wachsmann, Fabian; Schupfner, Martin; Legutke, Stephanie; Giorgetta, Marco; Reick, Christian; Gayler, Veronika; Haak, Helmuth; de Vrese, Philipp; Raddatz, Thomas; Esch, Monika; Mauritsen, Thorsten; von Storch, Jin-Song; Behrens, Jörg; Brovkin, Victor; Claussen, Martin; Crueger, Traute; Fast, Irina; Fiedler, Stephanie; Hagemann, Stefan; Hohenegger, Cathy; Jahns, Thomas; Kloster, Silvia; Kinne, Stefan; Lasslop, Gitta; Kornblueh, Luis; Marotzke, Jochem; Matei, Daniela; Meraner, Katharina; Mikolajewicz, Uwe; Modali, Kameswarrao; Müller, Wolfgang; Nabel, Julia; Notz, Dirk; Peters, Karsten; Pincus, Robert; Pohlmann, Holger; Pongratz, Julia; Rast, Sebastian; Schmidt, Hauke; Schnur, Reiner; Schulzweida, Uwe; Six, Katharina; Stevens, Bjorn; Voigt, Aiko; Roeckner, Erich (2019). MPI-M MPIESM1.2-HR model output prepared for CMIP6 CMIP. Earth System Grid Federation. doi:https://doi.org/10.22033/ESGF/CMIP6.741 .

Silvers, Levi; Blanton, Chris; McHugh, Colleen; John, Jasmin G; Radhakrishnan, Aparna; Rand, Kristopher; Balaji, V; Dupuis, Christopher; Durachta, Jeff; Guo, Huan; Hemler, Richard; Lin, Pu; Nikonov, Serguei; Paynter, David J; Ploshay, Jeffrey; Vahlenkamp, Hans; Wilson, Chandin; Wyman, Bruce; Robinson, Thomas; Zeng, Yujin; Zhao, Ming (2018). NOAA-GFDL GFDL-CM4 model output prepared for CMIP6 CFMIP. Earth System Grid Federation. doi:https://doi.org/10.22033/ESGF/CMIP6.1641

Swart, Neil Cameron; Cole, Jason N.S.; Kharin, Viatcheslav V.; Lazare, Mike; Scinocca, John F.; Gillett, Nathan P.; Anstey, James; Arora, Vivek; Christian, James R.; Jiao, Yanjun; Lee, Warren G.; Majaess, Fouad; Saenko, Oleg A.; Seiler, Christian; Seinen, Clint; Shao, Andrew; Solheim, Larry; von Salzen, Knut; Yang, Duo; Winter, Barbara; Sigmond, Michael (2019). CCCma CanESM5 model output prepared for CMIP6 C4MIP. Earth System Grid Federation. doi:https://doi.org/10.22033/ESGF/CMIP6.1301 .

Takemura, Toshihiko (2019). MIROC MIROC6 model output prepared for CMIP6 AerChemMIP. Earth System Grid Federation. doi:https://doi.org/10.22033/ESGF/CMIP6.9121

Volodin, Evgeny; Mortikov, Evgeny; Gritsun, Andrey; Lykossov, Vasily; Galin, Vener; Diansky, Nikolay; Gusev, Anatoly; Kostrykin, Sergey; Iakovlev, Nikolay; Shestakova,

Anna; Emelina, Svetlana (2019). INM INM-CM5-0 model output prepared for CMIP6 CMIP. Earth System Grid Federation. doi:https://doi.org/10.22033/ESGF/CMIP6.1423 .
Yukimoto, Seiji; Koshiro, Tsuyoshi; Kawai, Hideaki; Oshima, Naga; Yoshida, Kohei; Urakawa, Shogo; Tsujino, Hiroyuki; Deushi, Makoto; Tanaka, Taichu; Hosaka, Masahiro; Yoshimura, Hiromasa; Shindo, Eiki; Mizuta, Ryo; Ishii, Masayoshi; Obata, Atsushi; Adachi, Yukimasa (2019). MRI MRI-ESM2.0 model output prepared for CMIP6 AerChemMIP. Earth System Grid Federation. doi:https://doi.org/10.22033/ESGF/CMIP6.633 .
Zhang, Fang; Wu, Tongwen; Shi, Xueli; Li, Jianglong; Chu, Min; Liu, Qianxia; Yan, Jinghui; Ma, Qiang; Wei, Min (2019). BCC BCC-CSM2MR model output prepared for CMIP6 C4MIP. Earth System Grid Federation. doi:https://doi.org/10.22033/ESGF/CMIP6.1723 .
Ziehn, Tilo; Chamberlain, Matthew; Lenton, Andrew; Law, Rachel; Bodman, Roger; Dix, Martin; Mackallah, Chloe; Druken, Kelsey; Ridzwan, Syazwan Mohamed (2019). CSIRO ACCESS-ESM1.5 model output prepared for CMIP6 C4MIP. Earth System Grid Federation. doi:https://doi.org/10.22033/ESGF/CMIP6.2286
