# Supplementary material for: Projected climate-driven changes in pollen emission season length and magnitude over the continental United States
Source: Nat Commun. 2022 Mar 15;13:1234. doi: 10.1038/s41467-022-28764-0 (PMC8924258; doi:10.1038/s41467-022-28764-0)
Supplement: Supplementary file 2 — Reporting Summary [file 41467_2022_28764_MOESM2_ESM.pdf]

nature research

Corresponding author(s): Yingxiao Zhang
Allison Steiner

Last updated by author(s): Jan 16, 2022

Reporting Summary

Nature Research wishes to improve the reproducibility of the work that we publish. This form provides structure for consistency and transparency in reporting. For further information on Nature Research policies, see our Editorial Policies and the Editorial Policy Checklist.

Statistics

For all statistical analyses, confirm that the following items are present in the figure legend, table legend, main text, or Methods section.

n/a	Confirmed	
☐	☒	The exact sample size (n) for each experimental group/condition, given as a discrete number and unit of measurement
☐	☒	A statement on whether measurements were taken from distinct samples or whether the same sample was measured repeatedly
☐	☒	The statistical test(s) used AND whether they are one- or two-sided<br>*Only common tests should be described solely by name; describe more complex techniques in the Methods section.*
☐	☒	A description of all covariates tested
☒	☐	A description of any assumptions or corrections, such as tests of normality and adjustment for multiple comparisons
☐	☒	A full description of the statistical parameters including central tendency (e.g. means) or other basic estimates (e.g. regression coefficient) AND variation (e.g. standard deviation) or associated estimates of uncertainty (e.g. confidence intervals)
☐	☒	For null hypothesis testing, the test statistic (e.g. F, t, r) with confidence intervals, effect sizes, degrees of freedom and P value noted<br>*Give P values as exact values whenever suitable.*
☒	☐	For Bayesian analysis, information on the choice of priors and Markov chain Monte Carlo settings
☒	☐	For hierarchical and complex designs, identification of the appropriate level for tests and full reporting of outcomes
☒	☐	Estimates of effect sizes (e.g. Cohen's d, Pearson's r), indicating how they were calculated

Our web collection on statistics for biologists contains articles on many of the points above.

Software and code

Policy information about availability of computer code

Data collection	The pollen emission data in this study are produced using the updated Pollen Emission Model for Climate Models (PECM). Source code is available in GitHub (https://github.com/steiner-lab/pecm) under access code https://doi.org/10.5281/zenodo.5874177.
Data analysis	The data analysis are conducted using NCL 6.6.2 (www.ncl.ucar.edu), and the model sensitivity analysis is conducted using the Sensitivity Analysis Library (SALib) in Python 3 (https://salib.readthedocs.io/en/latest/).

For manuscripts utilizing custom algorithms or software that are central to the research but not yet described in published literature, software must be made available to editors and reviewers. We strongly encourage code deposition in a community repository (e.g. GitHub). See the Nature Research guidelines for submitting code & software for further information.

Data

Policy information about availability of data

All manuscripts must include a data availability statement. This statement should provide the following information, where applicable:
- Accession codes, unique identifiers, or web links for publicly available datasets
- A list of figures that have associated raw data
- A description of any restrictions on data availability

Observed historical pollen count data are from the National Allergy Bureau (NAB) of the American Academy of Allergy, Asthma and Immunology (AAAAI) (https://pollen.aaaai.org/#/).
Land cover data used in PECM simulations are from the Biogenic Emissions Landuse Dataset version 3 (BELD3), available at https://www.epa.gov/air-emissions-modeling/biogenic-emissions-landuse-database-version-3-beld3
Projected land cover change data for PFTs are from GCAM-Demeter land use dataset, available at https://data.pnnl.gov/group/nodes/dataset/13192
CMIP6 data are publicly available at https://esgf-node.llnl.gov/search/cmip6/

The processed data used to produce all figures in the manuscript are available from the UM Deep Blue Data Repository (https://doi.org/10.7302/628t-r416). The raw data of the simulated historical and future daily pollen emission for 15 CMIP6 models generated in this study have also been deposited in Deep Blue (www.deepblue.lib.umich.edu) under access code https://doi.org/10.7302/1s0g-b468

Field-specific reporting

Please select the one below that is the best fit for your research. If you are not sure, read the appropriate sections before making your selection.

☐ Life sciences ☐ Behavioural & social sciences ☒ Ecological, evolutionary & environmental sciences

For a reference copy of the document with all sections, see nature.com/documents/nr-reporting-summary-flat.pdf

Ecological, evolutionary & environmental sciences study design

All studies must disclose on these points even when the disclosure is negative.

Study description	This modeling study simulated both future and historical pollen emissions based on the PECM pollen emission code provided. The pollen model parameterizations are developed from observed pollen counts from the National Allergy Bureau of the American Academy of Allergy, Asthma and Immunology (AAAAI).
Research sample	Pollen emissions are generated based on daily meteorology data (e.g., temperature and precipitation) from a group of CMIP6 models. The CMIP6 meteorological data for different samples are available at https://esgf-node.llnl.gov/search/cmip6/
Sampling strategy	Data from 15 models are used in this study. We selected all of the available CMIP6 model data we could download at the time of the simulations.
Data collection	The research data are produced by Pollen Emission model for Climate Models (PECM; https://github.com/steiner-lab/pecm, DOI: https://doi.org/10.5281/zenodo.5874177). Yingxiao Zhang used Fortran to write all of the output data into NetCDF files.<br>The model is developed based on the pollen counts data from National Allergy Bureau (NAB) of the American Academy of Allergy, Asthma and Immunology (AAAAI), where pollen count data are collected by AAAAI member volunteers in a group of NAB pollen counting stations.
Timing and spatial scale	We simulated daily pollen emission for 20 years both in historical (1995-2014) and future (2081-2100) time periods. The 20-year period is a typical length for recent climatological averages. We regridded land cover and meteorological data to 25km resolution over continental United States for analysis.
Data exclusions	No data were excluded.
Reproducibility	The study is based on model simulations that can be reproduced from the provided code and data.
Randomization	n/a. We produced the data using model simulations. Due to the stochastic nature of climate model simulations and the comparison of multiple sensitivity test simulations, randomization in the study design is not necessary.
Blinding	n/a. All the data analyzed in this study are produced by the model we designed. When developing the model, the spatial and temporal limitations of the pollen count network precluded a blinded experiment.

Did the study involve field work? ☐ Yes ☒ No

Reporting for specific materials, systems and methods

We require information from authors about some types of materials, experimental systems and methods used in many studies. Here, indicate whether each material, system or method listed is relevant to your study. If you are not sure if a list item applies to your research, read the appropriate section before selecting a response.

Materials & experimental systems

n/a	Involved in the study	
☒	☐	Antibodies
☒	☐	Eukaryotic cell lines
☒	☐	Palaeontology and archaeology
☒	☐	Animals and other organisms
☒	☐	Human research participants
☒	☐	Clinical data
☒	☐	Dual use research of concern

Methods

n/a	Involved in the study	
☒	☐	ChIP-seq
☒	☐	Flow cytometry
☒	☐	MRI-based neuroimaging
